# Freehand pedicle screw fixation: A safe recipe for dorsal, lumbar and sacral spine

**DOI:** 10.12669/pjms.35.3.981

**Published:** 2019

**Authors:** Muhammad Junaid, Ali Afzal, Anisa Kalsoom, Syed Sarmad Bukhari

**Affiliations:** 1 *Dr. Muhammad Junaid, FCPS IFAANS, Department of Neurosurgery, PNS Shifa Hospital, Karachi, Pakistan*; 2 *Dr. Ali Afzal, FCPS, Department of Neurosurgery, Jinnah Postgraduate Medical Center, Karachi, Pakistan*; 3 *Dr. Anisa Kalsoom, FCPS, Department of Radiology, Fauji Foundation Hospital, Rawalpindi, Pakistan*; 4 *Dr. Syed Sarmad Bukhari, MBBS, Department of Neurological Surgery, Aga Khan University Hospital, Karachi, Pakistan*

**Keywords:** Freehand technique, Pedicle screw fixation, Spinal fracture

## Abstract

**Objective:**

To determine outcome of freehand pedicle screw fixation for dorsal, lumbar and sacral fractures at a tertiary care centre in the developing world.

**Methods:**

A retrospective review was performed of 150 consecutive patients who underwent pedicle screw fixation from January 1, 2012 to 31^st^ December 2017. A total of 751 pedicle screws were placed. Incidence and extent of cortical breach by misplaced pedicle screw was determined by review of intra-operative and post-operative radiographs and/or computed tomography.

**Results:**

Among the total 751 free hand placed pedicle screws, four screws (0.53%) were repositioned due to a misdirected trajectory towards the disc space. six screws (0.79%) were identified to have cause moderate breach while four screws (0.53%) cause severe breach. There was no occurrence of iatrogenic nerve root damage or violation of the spinal canal.

**Conclusion:**

Free hand pedicle screw placement based on external landmarks showed remarkable safety and accuracy in our center. The authors conclude that assiduous adherence to technique and preoperative planning is vital to success.

## INTRODUCTION

Pedicle screw fixation with rod constructs has attained global acceptance for stable spine fixation.^1^ A variety of techniques have been described in modern literature with free hand techniques having the unique advantage of being universally applicable, especially in the developing world with a dearth of equipment.^2^ These free-hand techniques rely heavily on a surgeon’s experience and ability to locate the pedicle entry point with anatomical landmarks. Freehand pedicle screw placement in the lumbar spine has enjoyed wide acceptance but screw placement in the thoracic spine is more challenging due to the critical regional neurovascular anatomy and the narrow pedicular corridor mandating higher accuracy and precision.^3^

The modern surgeon has excellent aides to enhance accuracy and safety of placement of pedicle screws in the spine which include intraoperative C-arm fluoroscopy, intraoperative computer tomography (CT), and computer-assisted navigation.^4^ Although these adjunct technologies have gained popularity, limitations include radiation exposure to the patient and surgeon as well as added high cost and prolonged operative times.^5^ Keeping in view the hazards of intraoperative radiation, the ability to place pedicle screws with anatomic landmarks alone are paramount.

Freehand pedicle screw placement in the thoracic spine is considered both safe and effective and routinely performed by many spine surgeons.^6^ However based on each surgeon’ straining and individual preferences there is no one single or uniform technique and considerable variations exist among studies that may not provide easily reproducible parameters. In addition, depending on the thoracic spinal level or region, many techniques have described varying entry points and trajectories.^7^ The study describes our step-by-step technique, which relies on a uniform entry point and sagittal trajectory for all levels.

## METHODS

The study was conducted at the department of Neurosurgery at PNS Shifa Hospital in Karachi, Pakistan after obtaining approval from the Institutional Review Board. The duration of study was five years from 1^st^ January 2012to 31^st^ December 2017 with a follow up of six months to one year. A retrospective review was performed of 150 consecutive patients who underwent pedicle screw fixation. A total of 751 pedicle screws were placed. This included patients of either sex, aged between 15 to 70 years. After taking history and doing clinical examination, a preoperative radiological assessment was done by using X rays anterio-Posterior and lateral views, MRI and CT scan with 3-D reconstruction of the lumbosacral spine. Patients above the age of 70 years, non-traumatic fractures, with associated disc herniation, previously operated and those who underwent fixation involving fluoroscopic guidance were excluded.

**Graph.1:**
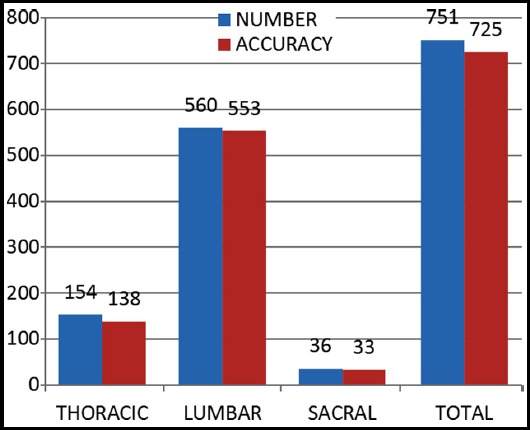
Screw accuracy per spinal level.

All the screws were inserted with free-hand technique using anatomic landmarks as a guide for an entry site. We used the following entry points: thoracic spine; 3 mm caudal to the junction of the transverse process and the lateral margin of the superior articulating process, and the sagittal trajectory was always orthogonal to the dorsal curvature of the spine at that level as described by Fennel et al, in lumbar spine; the junction of the pars interarticularis, the midpoint of the transverse process and the inferior point of the superior articular facet, in sacral; the infero-lateral margin of the basis of the superior articular process of the sacrum

We collected data on the number of patients, the number of inserted pedicle screws, and parameters of the surgical technique (entry point, axial, and sagittal trajectories). Incidence and extent of cortical breach by misplaced pedicle screw was determined by review of intra-operative and post-operative radiographs and/or computed tomography.

### Statistical analysis

Data was analyzed by using Microsoft Excel. Percentages and frequencies were calculated.

## RESULTS

**Fig.1:**
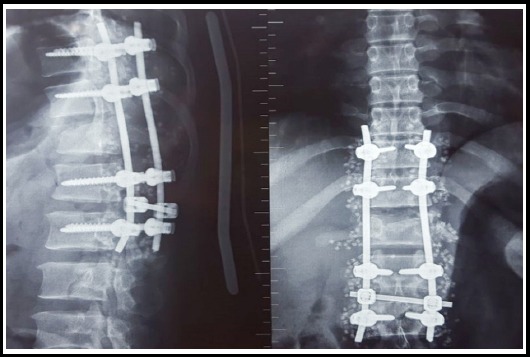
Plain X-ray thoracolumbar spine showing post-operative images of a D11-L3 fixation for a L1 compression fracture.

There were 107 (71.3%) males and 43 (28.7%) females with a mean age of 34.84 ± 14.6 years. The lumbar spine was the commonest site of placement with 561(74.6%) screws, followed by thoracic in 154(21.4%) and sacral in 36(4%). Among the total 751 free hand placed pedicle screws,12 screws (1.59%) were identified to have cause minor breach out of which nine had lateral breaches and 3 inferior breaches (Table-I). Minor breaches didn’t require repositioning and were only identified by postoperative imaging. There were no medial breaches while 8 screws (1.06%) caused major breach/misdirected and were identified peroperatively due to giving way feel of screws which were rectified there and then. 6(0.79%) screws had partially misdirected trajectory towards the disc space and were left undisturbed due to good individual holding strength and of the whole construct. No repositioning was done postoperatively. The overall accuracy of screw placement was 96.5% (Fig.1). There was no occurrence of iatrogenic nerve root damage or violation of the spinal canal or any vascular complication or CSF leak

**Table-I:** Screw characteristics.

Spinal level	Number	Accuracy	Breaches n=20				Misdirected n=6

			Major n= 8 (1.06%)	Minor n= 12 (1.59%)			

				Medial	Lateral	Inferior	
Thoracic	154	89.6%	5	0	6	2	3
Lumbar	560	98.7%	3	0	2	0	2
Sacral	36	91.7%	0	0	1	1	1

Total	751	96.5%	8	0	9	3	6

**Fig.2:**
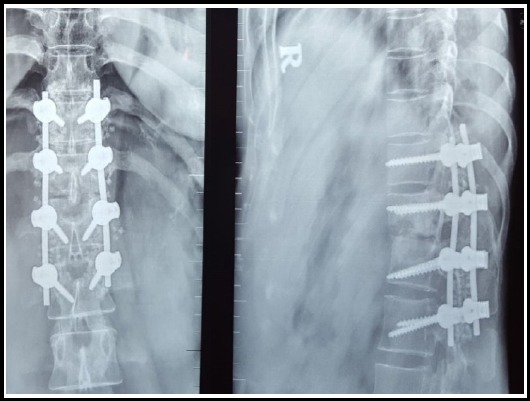
Plain X-ray thoracolumbar spine showing post-operative images of a D11-L2 fixation for a D12-L1 collapse.

## DISCUSSION

The placement of freehand pedicle screws especially in the dorsal spinal segment can be a challenge due to the varied and complex anatomy of the thoracic vertebrae, with a potential for screw malposition and neurovascular injury.^2^,^6^,^8^,^9^ Even though the modern spinal surgeon has various intraoperative navigational tools in his arsenal, sustained radiation exposure over one’s career is concerning. To avoid harmful effects of radiation, freehand pedicle screw placement is becoming the preferred modality of fixation for a diverse number of pathologies ranging from trauma to tumors.^10^ Command over freehand techniques based on basic anatomy is not only essential for budding surgeons but valuable in settings where availability of neuronavigation is limited.

**Fig.3:**
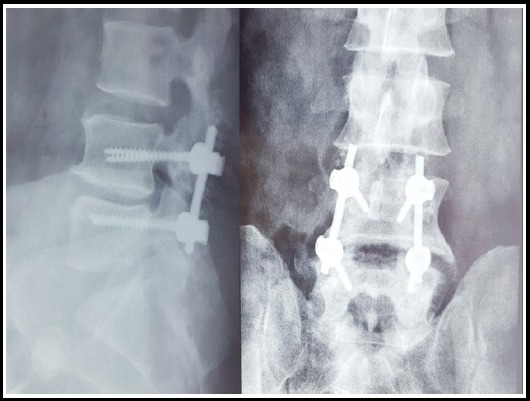
Plain X-ray thoracolumbar spine showing post-operative images of a L4-5 pedical screw fixation for dynamic listhesis.

The mean accuracy for placement of the screws in our patient group was 96.6%. Literature search reveals that over 8,000 screws have been placed in different studies with the mean accuracy for placement of the thoracic screws at 93.3% where the pedicle screw accuracy was defined as “having the entire screw contained within the cortices of each respective pedicle”.^11^ The overall mean accuracy rate for pedicle screws placement in studies was 93.34% (standard deviation of 3.54). The lowest reported accuracy was 87.4%^12^ in screws only at T1 and the highest was 98.3% in their series of 964 patients.^13^ Modi et al. calculated the difference inaccuracy for spine pathologies and found that the accuracy for patients with adolescent idiopathic scoliosis was 86.1%, patients with cerebral palsy - 91.7%, Duchenne’s muscular dystrophy - 95.9%, spinal muscular atrophy - 90.2%, and polio - 84.4%. The differences between diseases were not statistically significantly.^14^ In studies with multiple surgeons; the accuracy for five surgeons was 93.8%^15^ and for eight surgeons was 98.3%.^16^

Among the total 751 free hand placed pedicle screws, 12 screws (1.59%) were identified to have cause minor breach out of which 9 had lateral breaches and three inferior breaches. Minor breaches didn’t require repositioning and were only identified by postoperative imaging. In a study with a total of 720 screws inserted, 623 screws (86.5%) were perfect and 97 screws (13.5%) were misplaced.^17^ Among these, 39 screws (40.2%) were medial and 58 (59.8%) were lateral, which shows that the prevalence of lateral misplacement was more in comparison to medial misplacement with reported deviation of the screw <2 mm (Grade 1) and no misplacement in the inferior and superior.

Most studies agree on the defined “safe zone” ranging from 2 to 4 mm for pedicle screw breach of the vertebral bodies. This safe zone allows the medial or lateral wall breach by the screw without clinical consequences for the patient.^18^,^19^ Kim YW et al. defined a breach of < 2 mm as a “definite safe zone”, a breach of 2 to 4 mm a “probable safe zone”, and a breach of 4 to 8 mm as a “questionable safe zone”.^20^ In a study by Karapinar L et al., the majority of the breaches were labelled as minor (< 2 mm) in the thoracic spine.^21^ Overall, a lateral breach was more common than a medial breach of the vertebral bodies. There was a range of 2.5% to 21.6% of the screws for lateral breach and 1.7% to 13.2% of the screws for medial breach, with the majority falling below 5% of the screws.^22^,^23^

Increasing number of centres are now shifting towards robotic surgery and several studies comparing freehand conventional techniques for pedicle screw fixation robot-assisted (RA) have been published, but with equivocal results. A meta-analysis of five studies with 257 patients and 1105 screws revealed that there was no difference in the accuracy between robot-assisted and conventional freehand pedicle screw placement at the 0 mm grading criteria (RR 1.08, 95 % CI 0.86, 1.35), and at 2 mm grading criteria (RR 1.02, 95 % CI 0.68, 1.51).^24^,^25^ Further high-quality studies are required to unequivocally recommend one surgical technique over the other.

There was no complication encountered in our series which is consistent with the majority of studies in literature which reported no neurological or vascular complications following freehand placement of thoracic screws. The study by Kim, YW et al. gives the strongest evidence of the safety of this technique with their series of 3,204 screws with the longest follow-up (10 years) without any neurological or vascular complication reported.^19^ Parker et al.^4^ compared eight different surgeons and found an overall low complication rate in the over 3,000 screws placed again suggesting that the technique is still safe when used by different surgeons. The overall complication rate after freehand pedicle screw placement in the thoracic spine was low with a 4.3% incidence of durotomies in their case series which included both thoracic and lumbar freehand screw placement.^13^

Lastly, while freehand thoracic screw placement techniques are universally employed, there is dearth of published studies outlining the technical nuances which is further complicated by various starting points and/or trajectories for each level of the thoracic spine, making the adoption of one technique awkward to the spinal surgeon or trainee.

## CONCLUSION

Free hand pedicle screw placement based on external landmarks showed remarkable safety and accuracy in our centre. The authors conclude that experience and assiduous adherence to technique and preoperative planning is vital to success.

### Author`s Contribution

**MJ** conceived and designed the study.

**AA** did data collection and manuscript writing.

**AK** did statistical analysis & editing of manuscript.

**SSB** did review and final approval of manuscript.
